# Engineering etiology-aligned in vitro models of human vessels

**DOI:** 10.1038/s41378-026-01397-9

**Published:** 2026-07-24

**Authors:** Qi Li, Jiaxin Lin, Wenyu Zou, Jiangfeng You, Shuyuan Yu, Ziqi Gao, Huilong Du, Xinyi Shen, Jun Yin, Huayong Yang, Luqi Shen, Hongzhao Zhou

**Affiliations:** 1 School of Engineering, Hangzhou Normal University, Hangzhou, 311121 People’s Republic of China; 2 State Key Laboratory of Fluid Power and Mechatronic Systems, Zhejiang University, Hangzhou, 310058 People’s Republic of China; 3 School of Mechanical Engineering, Zhejiang University, Hangzhou, 310058 People’s Republic of China; 4 Women’s Hospital, Zhejiang University School of Medicine, Hangzhou, 310006 People’s Republic of China; 5 State Key Laboratory of Materials Processing and Die & Mould Technology, Huazhong University of Science and Technology, Wuhan, 430074 China

**Keywords:** Nanostructures, Electrical and electronic engineering

## Abstract

Vascular diseases remain a major global health burden, yet traditional animal models often fail to capture the human-specific mechanisms that drive disease progression. Recent policy shifts, including the FDA Modernization Act and the NIH’s transition away from animal-only studies, have intensified the need for human-relevant vascular platforms. This review introduces an etiology-to-model framework that maps six principal classes of vascular disease, including congenital, metabolic, neoplastic, inflammatory, degenerative, and risk factor-induced, to the in vitro systems best equipped to reproduce their defining microenvironmental disturbances. We evaluate how 2D assays, organoids, organ-on-chip platforms, tissue-engineered grafts, and bioprinted vessels each recapitulate distinct structural, cellular, and hemodynamic features of human pathology. We argue that the central challenge is no longer the lack of advanced tools, but the need to validate models against disease-specific benchmarks and integrate biological complexity without compromising reproducibility. By embedding disease etiology into model design, this framework provides a foundation for developing predictive vascular platforms that accelerate mechanistic insight and support precision therapy development.

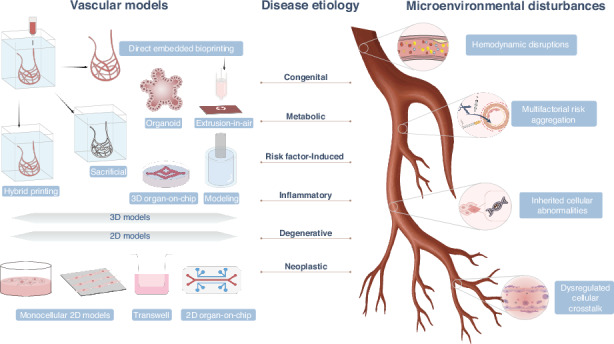

Cardiovascular and vascular diseases remain the leading cause of global mortality, accounting for nearly one-third of all deaths worldwide^1^. Although these disorders manifest in diverse forms, ranging from heart attack and stroke to diabetic retinopathy and vascular dementia^2^, they share a unifying root: the progressive loss of vascular homeostasis^3^. Blood vessels are not passive conduits but dynamic, multicellular tissues whose integrity depends on finely tuned interactions among genetic programs, cell-cell communication, extracellular matrix (ECM) remodeling, and biomechanical forces, such as shear stress and pressure. Disruption of this delicate balance initiates pathological cascades that evolve into congenital, metabolic, inflammatory, degenerative, neoplastic, or risk factor-induced diseases^4–6^.

Animal models have long underpinned vascular research, but persistent species differences in vessel architecture, cellular composition, and immune regulation limit their predictive value for human disease. The high attrition rate of vascular therapeutics in clinical trials reflects this translational gap^7^. In response, regulatory agencies are shifting decisively toward human-relevant systems. The US FDA Modernization Acts 2.0 and 3.0 now authorize non-animal platforms, including organ-on-chip models, for regulatory evaluation^8^. In 2025, the National Institutes of Health (NIH) announced a shift toward human-relevant new approach methodologies and reduced reliance on animal-only research^9^. Parallel movements are emerging internationally: the UK Government’s November 2025 roadmap outlines a phased reduction of animal testing across biomedical research, and similar discussions are accelerating within the EU^10^. Together, these shifts underscore an urgent need for in vitro vascular models (engineered platforms that replicate vascular structure and function) that faithfully recapitulate human physiology and pathophysiology.

Recent advances in bioengineering have enabled the development of such systems. Patient-derived or gene-edited cells, tunable ECM, and perfusion bioreactors enable in vitro models that reproduce critical aspects of vascular physiology and pathology^11,12^. Two-dimensional (2D) cultures support the dissection of single-cell mechanisms and enable high-throughput screening. Three-dimensional (3D) platforms, including organoids, organ-on-chip systems, tissue-engineered constructs, and bioprinted vessels, introduce structural fidelity, multicellularity, and dynamic flow^13,14^. These models are increasingly capable of reconstructing the genetic, cellular, and mechanical determinants of disease in a controlled human context.

However, despite this progress, a fundamental question remains unanswered: how should we match a disease’s etiology (primary genetic, metabolic, and mechanical drivers of vascular pathology) with the model best suited to study it? Current reviews often focus on individual technologies and their functions, but few provide a systematic framework that integrates the disease cause and the model. This review proposes an “etiology-to-model” framework that aligns six major classes of vascular disease with in vitro platforms optimized to capture their defining microenvironmental disturbances (Fig. 1). By linking congenital mutations, metabolic stress, oncogenic signaling, immune activation, degenerative remodeling, and environmental risk factors to the models capable of reproducing them, we outline a roadmap for precision in vitro vascular modeling. This framework not only clarifies how current technologies can be deployed but also highlights opportunities for innovation in a post-animal-testing era.

**Fig. 1:**
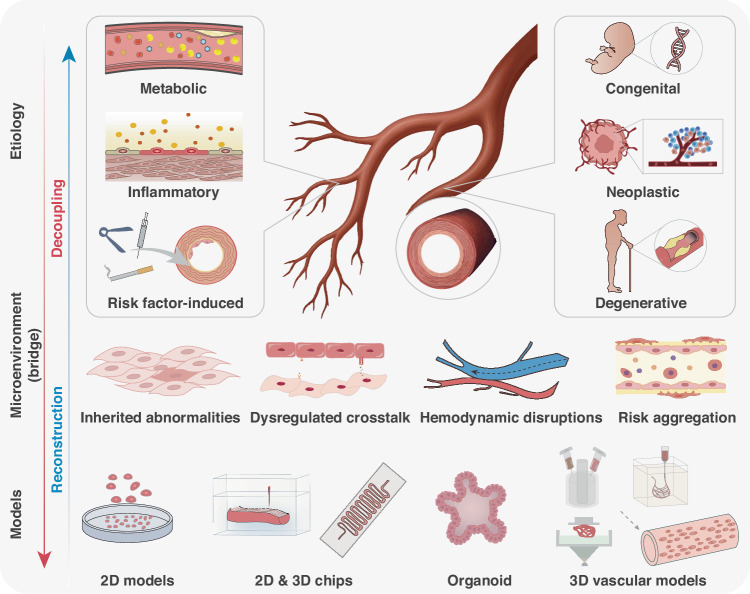
Engineering etiology-aligned in vitro models of human vessels. Schematic overview of the proposed framework linking disease etiology, microenvironmental disturbances, and corresponding vascular modeling strategies

## Blood vessel microenvironment in health and disease

Blood vessels are organized tissues where cells, extracellular matrix, and mechanical forces work together to maintain homeostasis. When these elements are disrupted, the vascular microenvironment breaks down, driving congenital, inflammatory, metabolic, degenerative, neoplastic, or risk factor-induced diseases. This section highlights how vascular architecture sustains health, how its failure triggers pathology, and why understanding this structure–function relationship is essential for designing in vitro models that faithfully recapitulate human disease.

### Blood vessel architecture across scales

Blood vessels are organized into arteries, arterioles, capillaries, venules, and veins, each adapted to specific roles via integrated microscale and macroscale structural features. For instance, arteries and veins manage long-distance transport and vasoactivity through three coordinated layers (intima, media, and adventitia), and arterioles and capillaries handle nutrient exchange via sparse endothelial cells (ECs).

Most vessels, aside from capillaries, contain three concentric layers: the tunica intima, tunica media, and tunica adventitia.

The tunica intima is a dynamic endothelial monolayer that senses hemodynamic forces, regulates barrier permeability, and maintains antithrombotic balance^15^. These functions rely on a network of molecules and pathways: Piezo1 and integrins convert shear stress and matrix tension into intracellular signals^16^; Syndecan-4 and Plexin D1 guide cell–matrix interactions^17^; E-selectin, P-selectin^18^, and the PI3K/Akt pathway govern leukocyte adhesion and barrier stability^19^. Integrity is its defining property^20^. In continuous endothelium such as the blood-brain barrier, tight junctions ensure selective transport^21^, while fenestrated or discontinuous capillaries enable rapid exchange in kidney, intestine, and liver^20^. In larger vessels, a cohesive intima prevents the entry of lipid and immune cells and thrombosis. Loss of integrity transforms the intima from gatekeeper to driver of pathology, emphasizing the necessity of reproducing flow-responsive endothelium, junctional stability, and the ability to shift between quiescent and activated states for in vitro models.

The tunica media provides contractility and compliance through vascular smooth muscle cells (VSMCs). In health, VSMCs maintain a contractile phenotype, responding to vasoactive signals via Ca²⁺-dependent pathways and cytoskeletal regulators such as RhoA/ROCK^22^. Under stress, VSMCs switch to synthetic or osteogenic states, driven by MAPK^23^, PKC^24^, and PI3K/Akt signaling^25^, promoting proliferation, ECM secretion, and calcification. For in vitro models, supporting phenotype switching under tunable mechanical load is essential to capture medial dysfunction in hypertension, atherosclerosis, and calcific vasculopathies.

The tunica adventitia contains fibroblasts, ECM proteins, vasa vasorum (supporting microvessels), and nervi vasorum (nerves). It reinforces structure, supplies nutrients to deeper layers, and regulates medial tone^26^. Collagen, making up ~64% of adventitial protein content, contributes to the mechanical modulus, ranging from ~4.2 MPa in veins to >16 MPa in arteries^27^. When wall thickness exceeds 0.5 mm, the vasa vasorum extends into the tunica media^28^. Adventitial fibroblasts actively remodel the ECM in response to injury^29^.

Across these layers, homeostasis also depends on interlayer communication: endothelial dysfunction alters VSMCs phenotype and decreases vascular tone^30^; medial stiffening perturbs endothelial shear sensing^31^; adventitial inflammation fuels medial remodeling^32^. Key signaling molecules include nitric oxide, prostaglandins, angiotensin II, endothelin, PDGF, TGF-β, and VEGF^33^. Thus, faithfully constructing the tunica layers in vitro is not a matter of anatomical mimicry but of re-establishing the functional crosstalk that determines whether vessels remain quiescent or progress toward pathology.

Except to these microscale architectures, blood vessels, vessel geometry and hemodynamics critically shape the vascular microenvironment. Diameters span from ~30 mm in the aorta to 5–10 μm in capillaries^34^, creating flow velocities from 40-50 cm/s in elastic arteries (shear stress: 25–43 dyn/cm²)^35^ to 0.03 cm/s in arterioles, where shear exceeds 100 dyn/cm² and stabilizes endothelium^36^. Branching follows Murray’s law (R^3^ = ∑r^3^, where R is parent radius, and r is daughter radius^37^), which minimizes energy loss^38^, but deviations at bifurcations generate disturbed and oscillatory shear that activate endothelium and trigger inflammation^34,39^. Vessel walls adapt to these demands: elastic arteries (1–2 mm thick) buffer pulsatile pressure, muscular arterioles (125–800 μm) regulate resistance^40^, and capillaries with ~0.5 μm walls maximize exchange^41^. Structural composition also shifts with function. ECM-rich conduits provide tensile strength, while muscularized vessels enable contractility^42^. These scale- and flow-dependent features explain why pathology localizes to specific sites (e.g., atherosclerotic plaques at branches) and highlight the need for in vitro models that integrate geometry, mechanics, and wall architecture to faithfully reproduce disease niches.

### Pathogenic drivers of vascular disease

Structure describes how vessels are built, and etiology categorizes the conditions that drive disease, but neither by itself reveals how normal vessels transition into pathology. To address this gap, we define “microenvironmental disturbance” as the characteristic disruption of structural, cellular, or mechanical balance within the vessel wall that converts a healthy architecture into a pathological one.

**Fig. 2:**
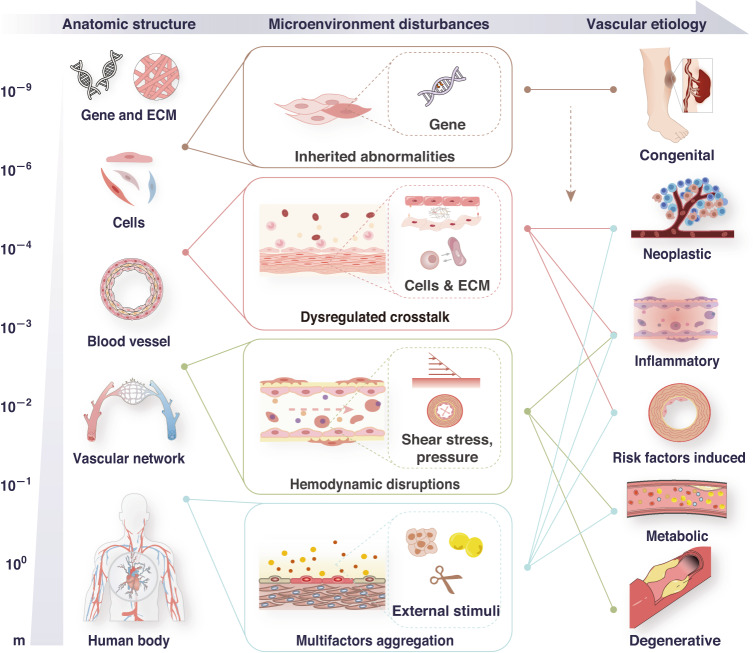
Blood vessel microenvironment in health and disease. Overview of the major cellular, extracellular, biochemical, and mechanical components of the vascular microenvironment in health and disease. The dashed line indicates that the inherited abnormalities may exacerbate other diseases

Inherited cellular abnormalities arise from mutations in genes that encode structural or signaling components of the vessel wall (Fig. 2a). Mutations in TIE2, FBN1, COL3A1, or NOTCH3 weaken endothelial junctions, elastic fibers, or mural cell support, creating fragile architectures that cannot withstand physiological stress^43^. These defects drive congenital vascular diseases such as Marfan syndrome, Ehlers-Danlos syndrome, and cerebral autosomal dominant arteriopathy (CADASIL)^44^, where the etiology is inseparable from the compromised structural integrity.

Dysregulated cellular crosstalk reflects breakdown in communication among ECs, VSMCs, pericytes, and fibroblasts (Fig. 2b). When inflammatory or metabolic signals disrupt these interactions, the vessel wall loses its coordinated remodeling capacity^45^. Endothelial barrier failure^46^, VSMC phenotypic switching^26^, and pericyte dropout collectively^47^ destabilize the microenvironment. This disturbance aligns with inflammatory, degenerative, neoplastic, and risk factor-induced etiologies, where pathology emerges less from a single cell defect and more from the collapse of intercellular coordination. In vitro modeling of such crosstalk requires multicellular systems that enable spatiotemporal analysis of interactions.

Hemodynamic disruptions demonstrate how geometry and mechanics dictate vulnerability (Fig. 2c). Laminar shear (5–50 dyn/cm², 5–10% strain)^48^ normally aligns and protects endothelium^49^, and modulates VSMC alignment, while oscillatory or excessive forces at bifurcations or under hypertension initiate pro-inflammatory signaling and thrombosis^50^. Hypertension^51,52^ and high pressure variability^53^ amplify wall tension and ECM deposition, promoting stiffening and aneurysm formation. Here, the related etiologies are degenerative and risk factor-induced diseases, originating not from faulty cells but from the structural context of flow and pressure that overwhelms them. Effective modeling must incorporate perfusion systems that simulate pulsatile flow, transmural pressure, and spatial shear gradients.

Multifactorial risk aggregation shows how external factors, including hyperglycemia^54^, dyslipidemia^55^, inflammation^56^, and oxidative stress^57^, interact with genetic or mechanical factors to erode vascular health (Fig. 2d). These insults rarely act alone; instead, they amplify one another, producing progressive endothelial dysfunction and metabolic vasculopathy that was commonly observed in diabetic vasculopathy and metabolic syndrome. Modeling requires long-term, perfusable platforms capable of reproducing metabolic gradients, immune activation, and redox stress.

Together, disease etiology manifests through microenvironmental disturbances, which often arise from structural vulnerabilities that can be recapitulated in vitro. These pathogenic drivers (mechanisms by which microenvironmental disturbances lead to vascular pathology) are interconnected: genetic predispositions amplify sensitivity to environmental or hemodynamic stress, while disrupted flow and chronic inflammation promote medial remodeling, calcification, and neoplastic transformation. In this context, in vitro vascular models, capable of precisely reproducing vessel architecture, multicellular crosstalk, and biomechanical forces, serve as indispensable translational platforms for dissecting pathogenesis and testing therapeutic strategies.

## In vitro models of vascular physiology and pathophysiology

In vitro vascular models span a spectrum of complexity, from 2D monolayers to multicellular bioprinted constructs—each offering unique insights but also distinct limitations. No single platform can replicate the full vascular microenvironment. Instead, the choice of system depends on which microenvironmental disturbance is most relevant to the disease being studied. Below, we outline major classes of models, highlighting their advantages and disadvantages in capturing structural, mechanical, and cellular dimensions of vascular pathology.

### Two-dimensional vascular models

2D systems remain the entry point for vascular biology because of their simplicity, reproducibility, and scalability. By culturing ECs or VSMCs on flat substrates, single drivers of disturbance with exceptional control could be isolated. 2D monolayers uncovered canonical mechanosensitive pathways such as Piezo1 and ion-channel regulation^58^, and enabled high-throughput therapeutic screens, for instance, identifying asiaticoside in hemorrhagic stroke^59^ and GSK3β inhibitors in Marfan syndrome^60^. Such studies demonstrate the unique strength of 2D systems in mapping cause–effect relationships that would be obscured in more complex environments.

Yet, single-cell simplicity comes at the cost of functionality. Pathology rarely arises from one cell type in isolation. To address this, co-culture systems pair ECs with VSMCs or immune cells, often on Transwells or lab-on-chip platforms. These multicellular arrangements allow the probing of paracrine signaling and shear-induced crosstalk, capturing processes such as PI3K/AKT or MAPK pathway activation^61^. Notably, these models have reproduced early atherogenic signaling under lipoprotein exposure^62^, as well as states of vascular quiescence^63^ and inflammation^64,65^ through EC-VSMC and EC-immune interactions.

Despite these advances, 2D systems ultimately remain confined to the plane. They cannot capture transmural mechanics, chronic remodeling, or the spatial gradients that define vascular physiology. Their enduring value is therefore conceptual: 2D models excel at dissecting the triggers of microenvironmental disturbance but cannot explain how these triggers integrate within a 3D, flow-adapted vessel wall. In this sense, they serve less as models of vascular disease than as “microscopes”, illuminating specific drivers that must later be tested in higher-order systems.

### 3D constructs and organoids

3D models mark a turning point in vascular research by restoring tissue-level architecture and dynamic cues that are absent in flat cultures. Unlike 2D systems, these constructs capture how vessel walls deform under flow, remodel in response to stiffening, or fail when multicellular coordination breaks down. Their common promise is fidelity to microenvironmental disturbances that arise only in 3D contexts: shear heterogeneity, matrix remodeling, and structural fragility.

Organ-on-chip systems, fabricated by lithography^66^, casting^67^, or laser bioprinting^68,69^, are among the most versatile. Microfluidic channels lined with endothelium reproduce physiological and disturbed flow regimes, making them powerful for dissecting hemodynamic disturbances^70^. These platforms allow real-time visualization of processes such as thrombolysis^71^, red blood cell deformation, and barrier modulation^70^. Disease-relevant extensions include neurovascular chips, where human induced pluripotent stem cell (iPSCs)-derived neurons combined with endothelial barriers replicate amyloid-β-induced inflammation in Alzheimer’s models^66^. Yet, despite their precision, chip systems often reduce complexity to single channels with minimal stromal or immune integration, limiting their ability to capture long-term degeneration or multicellular remodeling.

Vascular organoids embrace self-organization, generating capillary-like networks with ECs, pericytes, and basement membrane components. They excel at modeling cell-intrinsic and metabolic disturbances: under hyperglycemia, organoids thicken their basement membranes, faithfully reproducing diabetic microangiopathy^12^; NOTCH3-mutant mural cell organoids mimic CADASIL pathology^72^; SARS-CoV-2 exposure disrupts barrier function, ameliorated by soluble ACE2 treatment^11^; and replicating treatment of graphene oxide nanoscale for lipid and inflammation-induced atherosclerosis^73^. These systems highlight the capacity of organoids to reveal how genetic or metabolic insults destabilize the microenvironment. Still, their spherical, non-perfused geometry prevents the study of flow-driven disturbances or mechanical adaptation, leaving important aspects of vascular disease unexplored^74^.

Tissue-engineered constructs adopt a complementary strategy by producing tubular vessels from biomaterials, such as collagen or fibrin, encapsulated with defined layers of endothelial, mural, and stromal cells. These systems support controlled analysis of multicellular crosstalk and structural remodeling under pathological stimuli. Exposed to cytokines and monocytes, engineered vessels display endothelial activation, foam cell formation, and matrix degradation, which are hallmarks of early atherosclerosis^13,75^. They have also reproduced rare disease phenotypes, such as ECM accumulation in Hutchinson–Gilford progeria vessels derived from patient iPSCs^76^. By varying geometry, such as bends and bifurcations, they further show how anatomical features shape site-specific endothelial dysfunction^39^. However, recreating microvascular heterogeneity, achieving long-term perfusion, and scalability remain major hurdles.

Taken together, 3D constructs move vascular modeling beyond molecular triggers toward structural fidelity and dynamic function. They reveal how pathology emerges not from isolated signals but from the breakdown of integrated structure–function relationships. Chips, organoids, and engineered vessels each capture a different dimension of disturbance, such as hemodynamic, genetic/metabolic, or multicellular remodeling, providing complementary but incomplete views of disease. The challenge and opportunity ahead are therefore synthesis: integrating the precision of microfluidics, the biological relevance of organoids, and the architectural fidelity of engineered vessels into platforms that reconstruct the causal pathways of disease. In this light, 3D models are not an endpoint but a transitional stage, laying the foundation for new technologies that aim to unite anatomy, mechanics, and cellular interaction in a system.

### Bioprinted vascular models

Bioprinting sits at the intersection of architecture and biology, as it is the only approach that can simultaneously program vessel geometry, layering, and matrix composition^77,78^. This programmable control makes bioprinting uniquely suited to model structure-driven disturbances, where anatomy, wall composition, and transmural mechanics are primary drivers of pathology.

Extrusion-based approaches have enabled layered vessel fabrication by depositing cell-laden bioinks in concentric patterns^14^, which are ideally suited to replicate dysregulated cellular crosstalk. It can restore interfaces between ECs, VSMCs and fibroblasts to reproduce early atherosclerosis^79^ and enabling studies of mural-endothelial crosstalk under infection of SARS-CoV-2^80^. These constructs are valuable for probing how layered organization affects inflammatory triggers^80^ and for modeling occlusive responses^14^. However, in-air extrusion is constrained in curvature and microvascular resolution due to gravity and surface tension. Therefore, it models medium-to-large vessel wall failures better than capillary-level conditions.

Embedded bioprinting addresses those limits by printing into a supporting bath that temporarily stabilizes soft filaments, enabling complex branching, overhangs, and thinner walls construction^81–83^. This geometric freedom makes it the preferred strategy for reproducing hemodynamic disruptions, as it can generate bifurcated and curved channels that replicate the spatial shear gradients that drive endothelial activation and thrombosis. This approach has revealed how the printing process can induce VSMC phenotype switching and how mural cells facilitate EC growth^84^. Variations in printhead design and material composition have produced strategies such as aspiration-based assembly and interfacial coacervation, which allow the generation of multicellular spheroids modeling Mönckeberg’s sclerosis in vitro^85^, as well as seamless integration of printed filaments into curved or bifurcated arterial structures^86,87^.

Sacrificial printing, in which fugitive inks are evacuated to form perfusable channels, excels at creating dense microvascular networks that support organ-level perfusion and angiogenesis, including cardiac^88^ and hepatic^89^ models. Its primary strength lies in replicating multifactorial risk aggregation: the high-density, perfusable geometry supports oxygen and nutrient gradients, metabolic zonation, and accumulation of inflammatory mediators, making it the method of choice for diabetic vasculopathy and metabolic syndrome models that require long-term endothelial exposure to hyperglycemia, dyslipidemia, and oxidative stress. Yet these channels are typically lined with only a single layer of ECs, lacking the wall stratification required to model wall-centric vascular diseases^90,91^. Hence, sacrificial printing is unable to simulate dysregulated cellular crosstalk.

Hybrid strategies that treat both the bath and the bioink as active components are emerging as particularly promising. They enable perfusable tubes with stiffness gradients, spatial heterogeneity of cell types, and oxygen/nutrient zoning^81,92^. For instance, these strategies allow to simultaneously recapitulate hemodynamic disruptions by printing bioinks into crosslinkable baths with disease-relevant stiffness and curvature^93,94^ and multifactorial risk aggregation. Such models are well suited for reconstructing vascularized tissue microenvironments, enabling the exploration of reciprocal interactions between arteries and their surrounding tissue environment.

Collectively, the choice of bioprinting modality should be guided by the disturbance to be modeled: extrusion in-air and direct embedded printing for layered wall biology and medium-vessel mechanics; sacrificial methods for branching geometry, capillary density, and angiogenesis^95^; and hybrid approaches for constructing tubular structures with host-like tissue integration and microenvironmental gradients. The field’s next step is not incremental improvements in printer hardware but the development of disease-aligned toolchains, including standardized bioinks and validated perfusion protocols, which allow bioprinting to deliver actionable physiological insight.

### Flow-regulated vascular models

Perfusion is the defining feature that transforms static constructs into dynamic vascular models. Blood vessels are not passive tubes but structures constantly shaped by shear stress, cyclic strain, and transmural pressure. Many pathologies, from atherosclerosis to aneurysm, emerge when this mechanical regulation of the microenvironment is disturbed. Reproducing these forces in vitro is essential for studying how structural vulnerabilities translate into hemodynamic disturbances.

Evidence from in vitro platforms underscores this principle. Introducing laminar flow (0.1–10 dyn/cm²)^96^ to otherwise static endothelial cultures induces junctional alignment and quiescence^97^, while oscillatory (±10 dyn/cm²)^98^ or disturbed flow provokes NF-κB activation, and adhesion-molecule expression^99,100^. Perfused, multilayered constructs extend these findings, showing how pulsatile pressure (5–8% strain and 1–1.5 Pa shear) drives wall remodeling^51,101^ and how excessive stretching disrupts endothelial integrity, promoting apoptosis and detachment^102^. These outcomes demonstrate that perfusion is not simply additive but transformative: the same construct without flow behaves as a different biological system.

Biochemical gradients add a further layer of realism. Through spatially and temporally controlled perfusion, bioreactors can simulate gradients of growth factors, metabolic substrates, and inflammatory mediators^103^. Gradients of VEGF direct angiogenic sprouting, while counter-gradients induce lateral migration and vessel expansion^104^. Perfusion with oxidized LDL or inflammatory cytokines elicits hallmark features of atherosclerosis processes that can be partially rescued by restoring laminar flow^13^. High-glucose perfusion models diabetic vasculopathy^105^, while controlled oxygen gradients recreate ischemia-reperfusion injury^106,107^. Real-time monitoring using integrated sensors for pH, dissolved oxygen, or metabolites enhances the fidelity of these simulations^108^.

The advantages of perfused models lie in their ability to couple architecture with dynamic regulation, capturing the disturbances most relevant to vascular disease, including inflammation at low shear, aneurysm under elevated wall stress, and calcification under cyclic fatigue. But limitations remain: current pumps often generate non-physiological waveforms, long-term perfusion can destabilize soft constructs, and scaling from microvascular to arterial pressures remains unresolved^109^. Even more elusive is modeling the variability of human hemodynamics, for instance, the circadian blood pressure cycles, turbulent surges, or chronic fluctuations that accumulate damage over decades. Despite these hurdles, the necessity is clear: without perfusion, vascular models miss the fundamental driver of disease. The flow links geometry to biology, converting structural vulnerability into microenvironmental disturbance and, ultimately, etiology. Recognizing perfusion as a biological imperative rather than a technical option reframes how we judge the maturity of any vascular platform.

**Fig. 3:**
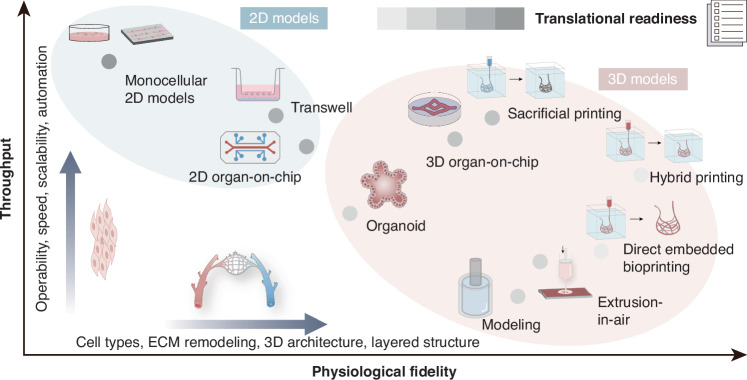
Evaluation of different disease models. Disease models (vascular models that incorporate specific pathological features to simulate clinical conditions) were evaluated from physiological fidelity (how well each model mimics human vascular structure and function, including cell types, ECM remodeling, 3D architecture, layered structure), throughput (how efficiently this model can be used at scale for experiments, involving operability, speed, scalability, automation), and translational readiness (how well the model connects to therapeutic pipelines, regarding standardization, reproducibility, regulatory acceptability, and predictive validity). The comparison provides a general framework for assessing model performance across different applications

Together, the progression from 2D cultures to 3D engineered constructs and advanced bioprinted models has markedly expanded our ability to replicate key features of vascular pathophysiology (Fig. 3). While early platforms laid the groundwork for dissecting cell-specific behaviors and signaling, newer models offer increasing spatial, mechanical, and biochemical complexity—enabling the study of emergent phenomena such as flow-driven remodeling, immune infiltration, and vessel wall failure. Bioprinting, in particular, introduces unprecedented control over architecture and microenvironmental heterogeneity, allowing more faithful reproduction of native and diseased vasculature (Table 1). When coupled with perfusion and dynamic modulation, these systems serve not only as static geometries but also as living, responsive systems for therapeutic development, positioning vascular modeling at the forefront of translational bioengineering.

**Table 1:** The evaluation of in vitro vascular models by dimension, advantages, limitations, and disturbance fidelity

Dimension	Models	Advantages	Limitations	Matched disturbance	Reference
2D	Single-cell monolayer	High throughput; Precise control of substrate stiffness; Ability to cell-intrinsic abnormalities analysis	Insufficient for reproducing multicellular interactions and vessel-wall mechanics	Inherited cellular abnormalities	^58–60,195,196^
	Multi-cell transwell/lab-on-chip	Captures paracrine signaling; Simple flow and early cellular crosstalk; Moderate throughput	Limited layering; Cannot reproduce full wall mechanics, geometry or flow-mediated disturbances	Inherited cellular abnormalities; Dysregulated cellular crosstalk	^61–65^
3D	Organ-on-chip	Perfusable lumen with tunable shear and strain; Support for mutifactorial stimulation; Moderate throughput	Simplified ECM; Limited remodeling and cell diversity	Hemodynamic disruptions; Multifactorial risk aggregation	^66–68,70,71,197,198^
	Organoids	Captures genetic defects and matrix interactions; Self-organization; Moderate throughput	Lack integrated perfusion; Nutrient/oxygen gradients; Weak mechanical cues	Inherited cellular abnormalities; Dysregulated cellular crosstalk	^12,73,74,199^
	Tissue-engineered grafts	Reconstructs integrated vessel-wall pathology for cellular, mechanical, and multicellular disturbances	Low throughput; Limited microvascular heterogeneity and scalability for complex multifactorial diseases	Inherited cellular abnormalities; Dysregulated cellular crosstalk; Hemodynamic disruptions; Multifactorial risk aggregation	^13,39,75,76,159,200^
	Extrusion in-air	Rapid assembly; Support for cellular crosstalk; Customizable geometry	High-viscosity inks can restrict growth; Resolution limited by gravity/surface tension	Dysregulated cellular crosstalk; Inherited cellular abnormalities; Hemodynamic Disruptions; Multifactorial Risk Aggregation	^14,79,80,85,201–203^
	Direct Embedded bioprinting	Variable spacial geometry; Multilayered vessel	Bath material optimization; Limited resolution	Inherited cellular abnormalities; Dysregulated cellular crosstalk; Hemodynamic disruptions; Multifactorial risk Aggregation	^82–84,86,87,204–208^
	Sacrificial bioprinting	Dense perfusable vascular networks; Oxygen gradient control; Angiogenesis	Limited diversity of cell types and ECM	Hemodynamic disruptions; Multifactorial risk Aggregation	^90,91,209–211^
	Hybrid bioprinting	Perfusable multi-layer vessels; Heterogeneity	Limited printing method; Scale-up difficult; Limited oxygen and nutrient	Inherited cellular abnormalities; Dysregulated cellular crosstalk; Hemodynamic disruptions; Multifactorial risk Aggregation	^81,92–94,212^

## Translating etiological insights into vascular models

Vascular diseases arise from diverse etiologies, but each expresses pathology through characteristic microenvironmental disturbances rooted in structural vulnerability. Understanding these links is crucial for designing models that move beyond generic vessel mimicry toward etiology-specific reconstructions of pathology. In this section, we highlight how representative in vitro systems have been applied to each disease class, what they reveal about the underlying disturbances, and where critical gaps remain (Table 2).

**Table 2:** Etiology-to-model mapping in vascular disease

Etiological Class	Exemplary diseases	Defining features	Microenvironmental disturbance	Representative models
Congenital	Marfan syndrome	• Genetic mutations (inherited / de novo)	• Malformed vascular architecture; • Defective endothelial differentiation; • Impaired vessel maturation and stabilization	• iPSC-derived 2D models^60,114^; • Perfused 3D organoids or grafts^72,76,115–117,168,213^
	CADASIL			
	HHT			
	Loeys-Dietz			
	HGPS			
Metabolic	Diabetes mellitus	• Metabolic dysregulation	• Endothelial metabolic stress; • Chronic low-grade inflammation; • Extracellular matrix glycation and stiffening	• 2D Transwell for ROS/lipid studies^122,129,214–217^; • Perfusable organ-chips^123^; • Coaxial bioprinted 3D vessels^12,94,124,125,218,219^
	Dyslipidemia			
	Atherosclerosis			
Neoplastic	Infantile hemangioma	• Dysregulated angiogenic signaling; • Tumor-driven vascular remodeling; • Oncogenic growth factor overactivation	• Pathological angiogenesis; • Tumor-vascular interactions; • Chaotic and leaky vascular networks	• 2D Transwell and organ-on-chip for drug screens^135,137,141,142,220^; • 3D organoids with ECM and stroma^136,138–140,143–145,221,222^
	Lymphangioma			
	Kaposi’s sarcoma and others			
Inflammatory	Takayasu arteritis	• Chronic vessel wall inflammation	• Endothelial activation; • Immune-cell infiltration and adhesion; • Pro-inflammatory cytokine milieu	• 2D monolayer cytokine assays^150,151,153–158^; • 3D perfusable immune-vascular chips^14,152,159^
	Kawasaki disease			
	Small-vessel vasculitis			
	Secondary vasculitis			
Degenerative	Vascular aging	• Aging-associated vascular degeneration; • Cellular senescence accumulation; • Oxidative stress-driven damage	• Extracellular matrix remodeling and degradation; • Reduced vascular elasticity; • Impaired repair and regeneration capacity	• 2D senescence and calcification screens^58,62,129^; • Biomechanically conditioned 3D grafts^13,>63,73,94,127–129^
	Vascular calcification			
Risk Factor-induced	Physical injury	• Chronic environmental/mechanical insults; • Disturbed flow dynamics; • Lifestyle-associated vascular stress	• Pathological hemodynamic remodeling; • Pro-inflammatory endothelial state	• 2D flow chambers^177,178,183,185,188,189,223^; • 3D bioprinted or organ-chip constructs^14,84,179,186,187,190–193,224–227^
	Biological insults			
	Chemical toxicants			
	Hypertensive remodeling			
	Disordered hemodynamics			

### Congenital vascular diseases

**Fig. 4:**
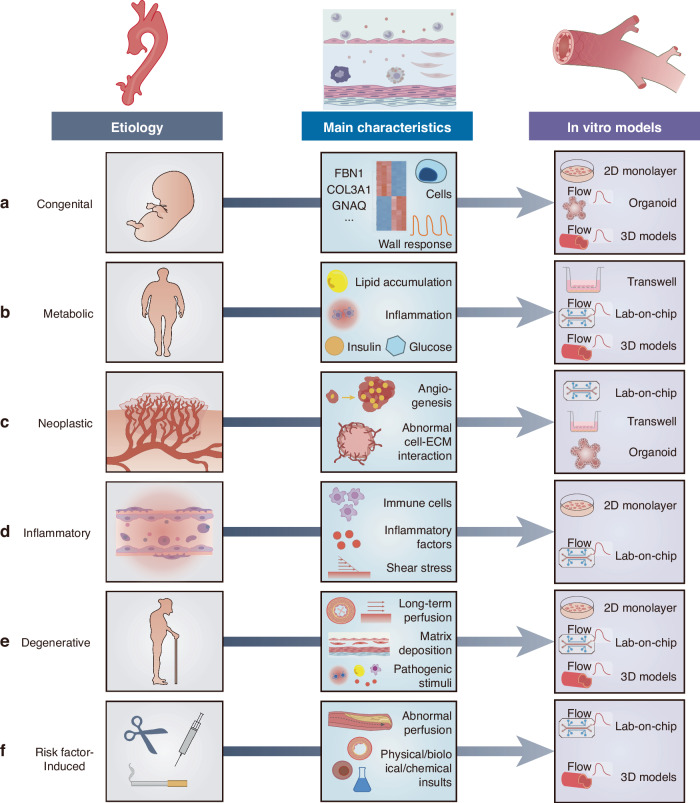
Applications of bioengineered vascular models in etiological disease classification. **a** Congenital vascular diseases. **b** Metabolic vascular diseases. **c** Neoplastic vascular diseases. **d** Inflammatory vascular diseases. **e** Degenerative vascular diseases. **f** Risk factor-induced vascular diseases. Representative modeling approaches are organized according to major etiological categories of vascular disease

Congenital vascular diseases comprise a heterogeneous group of disorders arising from abnormalities in vascular development, which may be driven by inherited or de novo mutations in genes that regulate vascular structure and function^110,111^. For instance, *FBN1* in Marfan syndrome, *COL3A1* in Ehlers-Danlos syndrome, *NOTCH3* in CADASIL, or *ENG* in hereditary hemorrhagic telangiectasia (HHT). These mutations create structural vulnerability, weakening extracellular scaffolds, mural support, or angiogenic signaling, which becomes pathogenic once exposed to hemodynamic stress (Fig. 4a)^111,112^. Therefore, in vitro models of congenital vascular diseases should reproduce how genetic defects disrupt vascular development, leading to structurally abnormal and unstable blood vessels.

2D monolayers of patient-derived or gene-edited ECs and VSMCs remain valuable for identifying cell-autonomous defects, such as impaired LDLR signaling^113^, defective angiogenesis in RNF213-mutant cells, or cytoskeletal abnormalities in Marfan VSMCs^60^. CADASIL-relevant NOTCH3 mutations reproduced junctional breakdown in neurovascular cocultures, supporting drug screening^114^. Yet, these planar systems lack the mechanical interplay that unmasks tissue-level failure.

3D organoids and engineered constructs address this limitation. NOTCH3-mutant organoids displayed mural cell dropout reversible by pharmacologic rescue^72^, while Marfan spheroids revealed altered fibrillin-1 mechanosensing^115^. Constructs incorporating Loeys-Dietz-specific TGFBR1 mutations recapitulated medial thickening and elastic fiber disarray^116^, while HGPS-derived vessels showed NOS3 dysregulation corrected by lonafarnib^76^. In HHT, microfluidic chips integrating pericytes uncovered flow-dependent leakage invisible in static culture^117^.

Hutchinson–Gilford Progeria Syndrome (HGPS) is a rare premature aging disorder caused by LMNA mutations that produce a protein, leading to vascular smooth muscle and endothelial cell dysfunction^118^. To study this, Atchison et al. developed patient-specific iPSC-derived tissue-engineered blood vessels. This 3D model allowed assessment of flow responsiveness, vasoconstriction, vasodilation, and inflammatory activation, recapitulating key HGPS vascular phenotypes and providing a physiologically relevant tool for dissecting endothelial contributions to HGPS vascular disease^76^.

In congenital vascular diseases, the contrast between 2D and 3D is not about superiority but suitability. 2D cultures of gene-edited or patient-derived cells remain indispensable for dissecting cell-autonomous defects, enabling high-throughput screens and precise pathway analysis. Yet, these platforms cannot reveal how mutations, such as FBN1 or NOTCH3, translate into wall fragility under flow. 3D organoids and engineered constructs add this missing dimension, exposing mural dropout, ECM failure, or shear-dependent leakage, while perfused systems recreate how genetic lesions unfold under mechanical stress.

### Metabolic vascular diseases

Metabolic vascular diseases are mainly caused by metabolic abnormalities, including hyperglycemia, insulin resistance, dyslipidemia and obesity-related inflammation^119,120^. These etiological factors disrupt vascular homeostasis by inducing characteristic microenvironmental disturbances, including dysregulated cellular crosstalk, hemodynamic disruptions and multifactorial risk aggregation (Fig. 4b)^5^. Consequently, modeling metabolic vascular diseases requires reproducing chronic metabolic stress, multicellular crosstalk, inflammatory activation, and long-term perfusion-dependent metabolic gradients.

Diabetes mellitus (DM) is characterized by chronic hyperglycemia caused by metabolic dysregulation (defects in insulin production, insulin action, or both)^121^. Diabetes models highlight how hyperglycemia disrupts barrier integrity. 2D monolayers reveal impaired insulin-NO signaling and Akt phosphorylation^122^, while 3D vascular organoids and neurovascular chips show that glucose, combined with cytokines, suppresses NOTCH^12^ and SIRT1^123^, reproducing diabetic microangiopathy. Organ-on-chip platforms incorporating pancreatic islets have simulated glycemic fluctuations and cytokine-driven endothelial injury, providing dynamic readouts of type 1 diabetes pathology^124^. 3D printed EC-microglia and embedded structures capture downstream consequences such as phenotype variation^125^, matrix stiffening and vascular remodeling^126^. Though diabetes mellitus is classified as a metabolic disease here, it should be noted that endothelial dysfunction, microvascular damage, and metabolic imbalance play important roles in the development of diabetes and its vascular complications as well^121^.

Dyslipidemia platforms demonstrate how lipid overload remodels the intima-media, contributing to the development of atherosclerosis. In vitro platforms have progressively advanced from reductionist assays^127^ to biomimetic constructs to capture this transition^94,128^. Transwell cocultures of ECs, VSMCs, and foam cells have clarified sequential pathways of cholesterol uptake and efflux, providing accessible insight into lipid handling^129^. Building on this, multilayered coaxially bioprinted vessels perfused with LDL and monocytes reproduce hallmark features of atherosclerosis—foam cell accumulation, intimal thickening, and matrix degradation—while enabling drug testing under physiologically relevant shear^94^. And iPSC-derived organoids exposed to lipid accumulation have even supported personalized screening with nanoscale therapeutics^73^.

Atherosclerosis is a chronic disease of medium and large arteries whose core feature is the accumulation of lipids within the arterial wall, leading to plaque formation^130^. Relevant studies have established pathological models that replicate the characteristics of the complex microenvironment of atherosclerosis. Zhang et al. developed a perfusable human tissue-engineered blood vessel (TEBV) model that reproduced early atherosclerotic features and supported therapeutic testing. While its humanized cellular composition and functional readouts enhanced physiological relevance, the straight-vessel geometry limited modeling of local hemodynamic disturbances^13^. More recently, advances in 3D bioprinting have enabled the incorporation of vascular geometry and hemodynamic regulation into atherosclerosis models. Li et al. developed an EoD bioprinted arterial model that reproduced key stages of atherosclerosis and incorporated pathological vascular geometries, enabling investigation of how disturbed flow cooperates with metabolic and inflammatory stimuli. This demonstrates the advantage of 3D bioprinting in capturing disease-relevant hemodynamic regulation beyond conventional straight-vessel tissue-engineered models. Furthermore, the model provided a versatile platform for dissecting the interplay between structural, hemodynamic, and inflammatory cues during atherosclerosis progression and for assessing therapeutic responses under physiologically relevant conditions, underscoring its value for disease mechanism studies and translational drug evaluation^131^. Although atherosclerosis is discussed here within the context of metabolic vascular diseases, it is also recognized as a multifactorial vascular disorder involving metabolic stress, chronic inflammation, immune-cell recruitment, extracellular matrix remodeling, and hemodynamic disturbances^132^.

In metabolic vascular diseases, 2D and 3D systems again serve complementary roles. 2D cultures remain powerful for high-throughput mapping of glucose- or lipid-sensitive pathways and for rapid drug screening. Yet, metabolic overload is inherently chronic and multicellular, involving fluctuating glucose, lipid deposition, and immune activation. 3D constructs and perfused chips, therefore, become essential to reveal how these biochemical disturbances translate into basement membrane thickening, foam cell formation, or vascular stiffening.

### Neoplastic vascular diseases

Neoplastic vascular diseases are distinguished by dysregulated growth and angiogenic signaling that promote pathological vascular remodeling and tumor progression^133^. These drivers disrupt vascular homeostasis by promoting dysregulated cellular crosstalk, hemodynamic disturbances, and multifactorial microenvironmental stress, resulting in pathological angiogenesis, aberrant vascular remodeling, and progressive vascular dysfunction^134^. Consequently, model selection should focus on the underlying disease mechanisms and the specific vascular features to be reproduced, including pathological angiogenesis, tumor-vascular interactions, and dysfunctional vascular architecture (Fig. 4c).

Benign tumors, including infantile hemangioma (IH) and lymphangioma, are featured with angiogenic and lymphangiogenic remodeling. In IH, 2D cultures of patient-derived ECs enabled high-throughput drug discovery, leading to propranolol as front-line therapy^135^. Yet only 3D spheroids and organoid platforms restored angiogenic sprouting and tissue-like gradients, allowing more predictive drug testing^136^. Similarly, lymphangioma models have progressed from 2D migration assays^137^ to 3D collagen cocultures^138^ and organ-on-chip platforms^139^, which capture interstitial flow-driven branching and remodeling^140^.

Malignant vascular tumors demand even greater fidelity. In Kaposi’s sarcoma (KS), 2D cultures of KSHV-infected ECs revealed STAT3-mediated inflammatory signaling but failed to maintain viral latency^141,142^. By contrast, 3D spheroids preserved KSHV genomes and enabled analysis of latency-associated PI3K/Akt/mTOR signaling^143^. A 3D rotating culture approach in Kaposiform hemangioendothelioma captures enhanced focal adhesion signaling and support sirolimus screening^144^. Aggressive entities such as angiosarcoma and epithelioid hemangioendothelioma benefit from 3D spheroid systems that replicate oncogenic fusion signaling and ECM-mediated drug resistance^145^, while 2D patient-derived monolayers remain essential for large-scale compound screening, such as romidepsin and 8-amino adenosine^146^.

Molley et al. developed a freeform bioprinting platform based on GelMA granular microgels, enabling the integration of perfusable endothelialized vessels, neoplastic cell aggregates, and stromal cells within a single 3D construct. The model successfully recapitulated tumor-vascular interactions, including distance-dependent tumor invasion and intravasation, while providing precise spatial control over the tumor microenvironment. Furthermore, co-culture experiments incorporating tumor cells, endothelial cells, and stromal cells confirmed the model’s ability to support physiologically relevant tumor-vascular crosstalk, angiogenic responses, and metastatic cell migration. By enabling the investigation of spatially regulated interactions between tumor, vascular, and stromal compartments within a biomimetic 3D environment, this platform provides a versatile tool for investigating cancer progression and assessing potential therapeutic strategies^147^.

In neoplastic vascular diseases, the key challenge is not simply modeling proliferation, but capturing context-specific cues, including viral latency in Kaposi’s sarcoma, hypoxia-driven angiogenesis in hemangioma, and fusion-driven signaling in angiosarcoma. 2D cultures remain valuable for rapid compound screening and pathway dissection, explaining their role in identifying propranolol for infantile hemangioma. Yet, 3D and perfused systems uniquely preserve tumor microenvironments where viral genomes persist, angiogenic sprouting occurs, or ECM-mediated resistance emerges.

### Inflammatory vascular diseases

Inflammatory vascular diseases are characterized by chronic vessel-wall inflammation^148^. The resulting vascular microenvironment is marked by dysregulated cellular crosstalk, hemodynamic disruptions and multifactorial risk aggregation, specifically manifested by endothelial activation, immune-cell recruitment, extracellular matrix remodeling, and loss of vascular homeostasis^149^. Therefore, an appropriate in vitro model should have the ability to capture multicellular interactions, dynamic microenvironmental regulation, and multifactorial disease progression (Fig. 4d).

Immune cell-driven infiltration and remodeling is exemplified by Takayasu arteritis, where macrophages and T cells infiltrate the adventitia, driving fibrosis and wall thickening. In vitro, patient-derived macrophages reveal IFN-γ-induced M1 polarization via glycolytic reprogramming and PFKFB3 activation^150^, while macrophage-aortic fibroblast cocultures uncover GPNMB-αvβ1 integrin-mediated fibrotic signaling^151^. 3D PEG-based microvessels embedding ECs, stromal cells, and monocytes under TLR7/8 stimulation recreate monocyte-mediated vascular disruption^152^, offering platforms to probe these diseases.

Cytokine- and serum-mediated endothelial injury dominates disorders such as Kawasaki disease and lupus vasculitis. Endothelial monolayers exposed to patient serum or TNF-α demonstrate junctional disruption and barrier loss, reversible by pathway-targeted inhibitors (USP5/NFATC1/TLR4 signaling)^153,154^. In parallel, bioprinted vascular models reveal that EC confluence and VSMC-EC interaction are critical for clotting resistance under inflammatory stress^14^. Patient immune-cell-monocyte co-culture 2D models have shown that PSGL-1 and GPIbα engagement activates TGF-β1 signaling, exacerbating coronary artery involvement^155^. Similarly, iPSC-derived vascular cells from lupus patients reproduce disease phenotypes for mechanistic dissection^156^.

Autoantibody- and neutrophil-mediated acute effector damage underlies ANCA-associated vasculitis. Neutrophil cultures stimulated with patient sera generate reactive oxygen species and neutrophil extracellular traps (NETs)^157^, while co-culture with dendritic or T cells recapitulates IL-6-driven immune amplification^158^. Advanced 3D microvessel systems reveal how transmural immune infiltration and oxidative stress collectively destabilize vascular integrity, enabling evaluation of targeted antioxidants and anti-inflammatory therapies^159^.

A representative example of modeling the inflammatory vascular microenvironment is the multicellular 3D bioprinted vessel developed by Gold et al. They simulated inflammatory vascular injury, demonstrating that interactions between ECs and VSMCs reduced inflammatory cytokine production and enhanced vascular protective signaling. This study illustrates how 3D bioprinting can bridge the gap between conventional endothelial monolayers and more physiologically relevant inflammatory vascular microenvironments^14^.

Inflammatory vascular diseases highlight how pathology stems from immune-vascular interactions rather than structure alone. 2D cultures excel at isolating cytokine effects, antibody signaling, and neutrophil activation, offering throughput and mechanistic clarity. Yet these systems cannot reproduce the layered infiltration, fibrosis, or chronic remodeling that define vasculitis. 3D and perfused models add this missing dimension, capturing how immune cells reshape the vessel wall under flow. Therefore, 2D and 3D are not alternatives but sequential tools that 2D identifies critical immune triggers and 3D further validates the signal evolve process.

### Degenerative vascular diseases

In this review, degenerative vascular diseases refer to vascular disorders characterized by progressive deterioration of vascular structure. Aging-associated processes, including cellular senescence, oxidative stress, and extracellular matrix remodeling, are key drivers of this degenerative process^160–162^. Effective modeling must therefore reproduce long-term aging processes, where cell-autonomous decline interacts with biomechanical stress.

Senescence represents a primary driver. ECs and VSMCs subjected to chronic oxidative stress lose proliferative capacity, disrupt NO signaling, and adopt a pro-inflammatory secretory phenotype. These changes not only impair vascular tone but also accelerate extracellular matrix degradation, predisposing to stiffening. In vitro, 2D cultures enable rapid induction of senescence by introducing assays (bleomycin for VSMCs^163^, bisphenol S^164^, advanced oxidation protein products^165^, and H_2_O_2_ for ECs^166^), and screening of modulators, while 3D lab-on-chip models incorporating senescent fibroblasts reproduce ECM stiffening and aberrant angiogenesis characteristic of the senescence-associated secretory phenotype^167^. Similarly, the application of HGPS patients' iPSC-derived VSMCs and cyclic strain results in a “progeria-on-chip” model, reproducing amplified inflammation and DNA damage and revealing the synergy between mechanical forces and genetic vulnerability^168^. In engineered vascular tissues, overexpression of SIRT6 in VSMCs mitigated the senescence, reducing inflammation and attenuating features of aneurysmal remodeling^169^.

To systematically investigate the cellular mechanisms underlying vascular aging, Zhang et al. generated a single-cell transcriptomic atlas of primate arteries using young and aged cynomolgus monkeys. Functional studies further demonstrated that FOXO3A is a central regulator of vascular homeostasis. This multi-scale platform combining in vivo aging models, single-cell profiling, and gene-editing-based validation provides a comprehensive framework for dissecting molecular drivers of vascular aging and age-associated vascular diseases^170^.

Vascular calcification is a defining feature of degeneration, driving stiffening and loss of compliance. 2D cultures of VSMCs under phosphate elucidated BMP2 signaling and downstream fibrotic and apoptotic responses^171^, and enabled screening of anti-calcification agents^172^, but oversimplify the process. 3D scaffolds and hydrogels provide added fidelity, showing how ECM cues guide transdifferentiation and mineral deposition. For example, VSMCs were encapsulated in hydrogels, showing transdifferentiation into a mineralizing phenotype via regulating hydrogel component^173^, which could be diminished by the silencing of α-SMA^174^. These models demonstrate that 3D context is essential for capturing the spatial and stepwise nature of pathological mineralization.

Degenerative vascular diseases demand models that can reconcile the slow tempo of aging with the mechanical and biochemical stresses driving vessel wall failure. Thus, long-term culture under cyclic strain and pulsatile flow is essential. 2D models facilitate rapid senescence induction and drug screening but oversimplify matrix remodeling. By contrast, 3D engineered constructs, especially those incorporating cyclic strain, long-term perfusion, and multicellular architecture, offer the structural and mechanical fidelity necessary to capture progressive degeneration.

### Risk factor-induced vascular diseases

Risk factor-induced vascular diseases are primarily defined by chronic exogenous perturbations of the vascular microenvironment. Diverse stimuli such as disturbed flow, hypertension, and toxic exposures converge on endothelial dysfunction and inflammatory activation, suggesting that diverse risk factors act on a common endothelial interface driving inflammatory responses (Fig. 4f)^175,176^. Their study requires platforms that allow precise, tunable control over hemodynamic and chemical stresses.

Elevated pressure and shear stress remodel vessel walls by promoting fibrosis, VSMC proliferation, and EC dysfunction. 2D pressurized fibroblast cultures replicate fibrotic responses associated with venous hypertension^177^, and ECs monolayers reveal that elevated shear disrupts homeostatic signaling networks, such as BMPR2, ERG, TGFBR2^178^. Furthermore, 3D DLP-printed adventitial constructs regulated the hydrogel component to control the stiffness, which could reproduce matrix stiffening under pressurization, showing the increased VSMC proliferation and α-SMA expression^179^.

Hypertension is a disorder primarily characterized by persistently elevated arterial blood pressure. In patient-specific cerebrovascular models reconstructed from clinical imaging, Epshtein et al. showed that coupling glycoprotein VI (GPVI)-mediated adhesion with aneurysm-specific hemodynamics could preferentially localize particles in rupture-prone regions. This approach provides a foundation for risk stratification and targeted intervention in cerebral aneurysms^180^. Although classified here within the risk factor-induced framework, hypertension is recognized as a multifactorial disorder, whose etiology involves a complex interplay between environmental factors such as high dietary sodium intake, obesity and psychosocial stress, and pathophysiological mechanisms including abnormalities in renal sodium handling, vascular remodeling, and endothelial dysfunction^180,181^.

Disordered flow conditions, such as stenosis, bifurcation, or turbulence, further compound vascular injury by triggering platelet activation and thrombosis. Thrombosis-on-a-chip models, incorporating arterial shear profiles and vascular geometry^182^, have enabled real-time quantification of clot formation^183^ and pharmacological response^184^. These outperform static 2D assays, where lesion-specific aggregation is observed but lacks spatial fidelity. Perfusable PDMS channels with axially aligned ECs and circumferentially aligned VSMCs offer an anatomically relevant benchmark, reinforcing the importance of geometrical and biomechanical accuracy^185^.

Physical and environmental insults compromise vascular stability through direct structural disruption and biochemical stress. Organ-on-chip systems simulating spaceflight or irradiation demonstrate junctional breakdown, increased permeability, or delayed senescence^186^, often unobservable in static culture. Similarly, layered bioprinted models reveal the critical role of multicellular architecture in mitigating injury-induced inflammation and thrombosis, highlighting the importance of multicellular design^14,84^. Chemical exposures, including nicotine^57^, uremic solutes^187^, PM2.5^188^, and plastics^189^, trigger oxidative stress, adhesion-molecule upregulation, and endothelial senescence in 2D assays, which are useful for pathway discovery. Yet these platforms overlook flow-sensitive pathology. 3D perfused chips recapitulate flow-sensitive effects, such as junctional disruption, oxidative-stress markers release^190,191^, and barrier leakage^187^, with sufficient physiological accuracy. Comparable systems also demonstrated fungal translocation across endothelial barriers^192^ and venom-induced delamination^193^, reinforcing the value of biomimetic design.

Together, these studies highlight a recurring principle: 2D models excel at isolating stimulus-specific pathways and enabling high-throughput drug testing, while 3D and perfused constructs capture how risk factors reshape vascular architecture under dynamic conditions. The integration of both approaches is essential—not only to dissect the immediate molecular triggers of injury but also to follow their structural consequences, bridging short-term perturbations with long-term vascular pathology.

Translating disease etiology into in vitro models makes clear that no single platform can capture the full spectrum of vascular pathology. 2D cultures remain invaluable for congenital and metabolic disorders where high-throughput screening and pathway-specific defects can be isolated. 3D organoids and tissue-engineered constructs excel in capturing multicellular crosstalk, making them well suited for metabolic, inflammatory, and neoplastic diseases that hinge on paracrine signaling and remodeling. Organ-on-chip systems recreate perfusion, barrier dynamics, and immune infiltration, essential for inflammatory and risk factor-induced vasculopathies. Bioprinted vessels uniquely reproduce layered geometry and tunable biomechanics, providing unmatched power for degenerative and large vessel diseases. Each platform offers distinct insights into microenvironmental disturbances, and together they form a complementary toolkit. The challenge and opportunity lie in selecting or integrating models according to the disturbance most central to the etiology under investigation.

## Challenges in clinical application and future perspectives

In vitro vascular platforms have reached impressive technical maturity, but that maturity has not translated into commensurate clinical impact. The central failing is not the deficiency in innovation or the absence of appropriate tools. It is the absence of a disciplined, etiology-driven approach to validation and deployment. To transform prototypes into clinically useful tools, the community must reframe priorities around benchmarks, integration, and reproducibility.

**Fig. 5:**
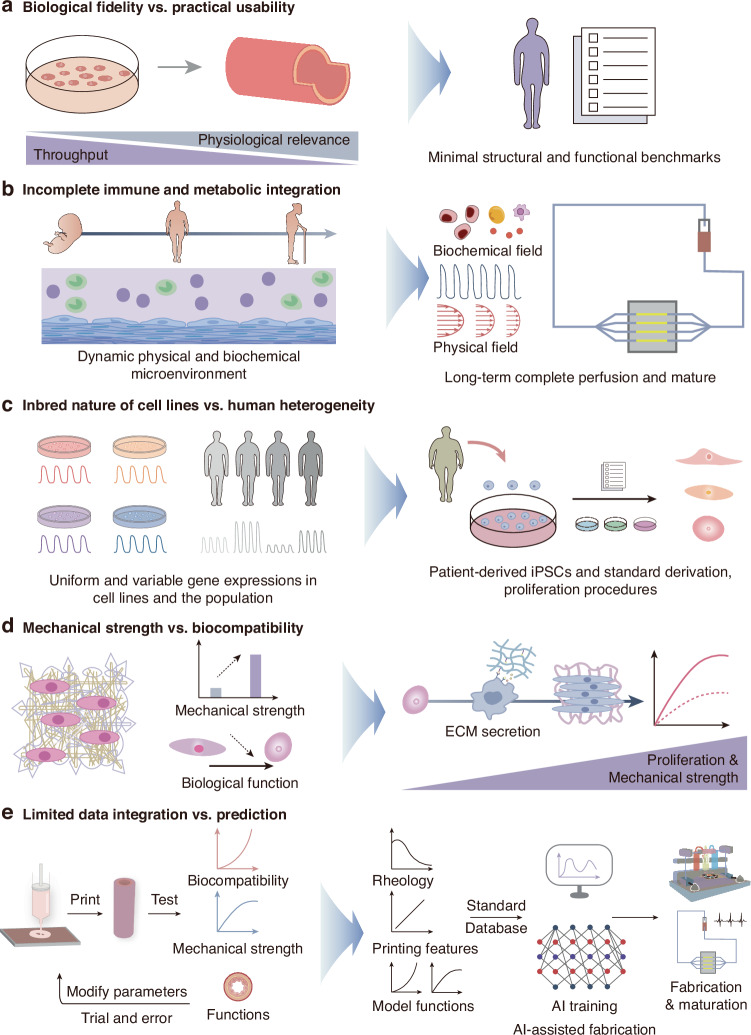
Major obstacles and opportunities in vascular tissue engineering. **a** Fidelity versus usability. **b** Incomplete immune and metabolic integration. **c** Genetic and patient specificity. **d** Mechanics and matrix remodeling. **e** Standardization, scalability, and data integration. Summary of key factors influencing the development and translation of engineered vascular models

First, validation must be function-first and disease-specific (Fig. 5a). A model’s value is not measured by physiological fidelity but by whether it reproduces the microenvironmental disturbance central to an etiology, such as oscillatory shear-driven endothelial activation for atherosclerosis, mural dropout under stress for CADASIL, and chronic hyperglycemic flux for diabetic microangiopathy. Defining compact, quantitative benchmarks from a set of agreed readouts, for instance, shear-responsive NF-κB activation, transmural permeability, and ECM elasticity, will optimize designs, facilitate cross-lab comparison, and provide regulators with concrete acceptance criteria. Equally important, these readouts should be connected to clinically meaningful endpoints to ensure that model performance can be interpreted in a disease-relevant context. For example, endothelial permeability quantified by 70-kDa FITC-dextran or fluorescent albumin leakage can be correlated with vascular leakage observed in diabetic retinopathy and inflammatory vasculitis; platelet adhesion, fibrin deposition, and thrombus occlusion time measured in thrombosis-on-chip systems can reflect clinical thrombotic risk; collagen degradation, elastin fragmentation, matrix metalloproteinase (MMP-2/MMP-9) activity, and vessel-wall compliance can serve as surrogate markers of aneurysm wall degeneration; calcium deposition quantified by Alizarin Red staining, calcium content assays, or osteogenic marker expression (RUNX2, BMP2) can be linked to vascular calcification detected by computed tomography angiography (CTA) imaging; and lipid accumulation, macrophage infiltration, and fibrous-cap thickness in engineered atherosclerotic vessels can be related to plaque vulnerability identified by intravascular ultrasound (IVUS), optical coherence tomography (OCT), or magnetic resonance imaging (MRI).

Beyond establishing validation benchmarks that reflect disease mechanisms, dynamic biological integration remains incomplete (Fig. 5b). Immune cells, long-term metabolic cycles, and microbial challenges are core drivers of many vascular diseases, rather than optional additives. Current systems, however, often reduce these disturbances to oversimplified surrogates and short-term perfusion, such as static cytokine dosing or glucose spikes. Existing obstacles, including sterility under long-term perfusion, immune-cell viability, and realistic metabolic cycling, could be solved by closed-loop perfusion systems, multi-organ platforms, and integrated real-time biosensors. Paying more attention to sterile long-term culture hardware and validated immune-cell sourcing will convert short-term snapshots into long-term disease trajectories.

Genetic and patient specificity also remain elusive (Fig. 5c). While immortalized lines dominate for convenience, they obscure the heterogeneity that drives clinical variability. iPSCs hold promise but suffer from inconsistent differentiation and immature phenotypes. For congenital diseases such as CADASIL or Marfan syndrome, where precise genetic lesions dictate pathology, such limitations are especially damaging. More rigorous pipelines combining CRISPR/Cas9 editing, isogenic controls, and single-cell transcriptomic quality checks will be essential. Building patient-derived iPSC biobanks linked with clinical metadata could transform the field by anchoring vascular models in real-world heterogeneity and therapeutic response. Integration of patient-derived cells with clinical imaging, genomic sequencing, transcriptomic profiling, and longitudinal outcome data would further enable model stratification according to disease stage and therapeutic responsiveness. For example, aneurysm-derived VSMCs may be paired with patient-specific CTA measurements of aneurysm growth, while CADASIL iPSC models may be compared with MRI white-matter lesion burden. Such approaches would allow in vitro readouts to serve as predictive biomarkers rather than isolated laboratory measurements. Achieving this goal will require engineered systems that can also capture the biomechanical and matrix-remodeling processes that influence disease progression.

Engineering challenges remain a major barrier to faithfully modeling vascular biomechanics and matrix remodeling (Fig. 5d). Native vessels are not static conduits: they display nonlinear elasticity, undergo cyclic strain, and dynamically remodel their ECM. These properties are central to pathologies, such as aneurysm formation, vascular stiffening, and calcification, yet most engineered constructs fail to reproduce them. Soft bioinks sustain cell growth but lack the resilience to endure mechanical load, while stiffer scaffolds provide stability at the expense of remodeling capacity. A promising strategy is to design materials that balance biological remodeling with mechanical integrity, and to develop long-term bioreactors that apply cyclic strain, transmural pressure, and shear. In other words, the goal is not to print the final wall but to create an environment in which cells rebuild the wall themselves, guided by mechanical cues.

Finally, reproducibility and data infrastructure are the bottlenecks to scale (Fig. 5e). Variability in cell sourcing, bioink formulation, printing protocols, and perfusion designs produce models that are difficult to compare across laboratories. This undermines not only scientific comparability but also regulatory acceptance, since no consensus exists on what constitutes a “validated” vascular model. Standardized datasets capturing fabrication parameters, performance metrics, and biological readouts are therefore indispensable. Future datasets should also incorporate clinically relevant reference standards, including OCT, IVUS, CTA, MRI, and angiography-derived measurements, circulating biomarkers, and patient omics profiles. Establishing such multiple benchmarks would enable direct comparison between model outputs and clinical disease manifestations, thereby strengthening regulatory confidence and translational utility while also providing a robust data foundation for computational modeling and optimization supported by artificial intelligence (AI). Once such repositories are established, computational tools, including AI and reinforcement learning, can play a transformative role: identifying hidden relationships between input parameters and construct fidelity, predicting optimal bioink formulations, and dynamically adjusting printing conditions in real time. In this way, the utility of AI ultimately depends on the availability of rigorous, shared data infrastructures.

Taken together, these priorities point to a single conclusion: the strength of in vitro vascular modeling lies not in mimicking intact vessels, but in the programmable reconstruction of the specific disturbances that drive disease. 2D assays enable rapid mechanistic screening; organoids and engineered tissues reveal multicellular remodeling; organ-on-chip systems integrate flow and immune dynamics; bioprinted constructs recreate structural failure modes. If benchmarks are standardized, immune and metabolic complexities are integrated, patient-centered cell sourcing is established, culture conditions are optimized, and data are shared, in vitro vascular models will finally bridge the gap between molecular insight and clinical translation.

## Conclusion

Vascular disease modeling is entering a decisive transition. Animal systems, long the default, have repeatedly failed to predict human outcomes, while policy shifts from the FDA Modernization Acts^194^ to the NIH’s 2025 move away from animal-only studies^9^, and the UK’s roadmap to reduce animal testing, now accelerate the adoption of human-relevant platforms^10^. In this review, we propose an “Etiology-to-model framework” that anchors model design in the root causes of disease, linking genetic, cellular communication, hydrodynamic, and aggregation disturbances to the in vitro platforms best suited to capture them. This perspective reframes models not as generic vessels, but as tools calibrated to specific pathological mechanisms: rapid 2D assays for molecular triggers, layered 3D constructs for structural degeneration, perfusable chips for hemodynamic remodeling, and bioprinted systems for wall-related failures. By embedding etiology into model choice, the field can move from descriptive mimicry toward predictive, precision-guided interventions. Challenges remain, including standardization, long-term dynamics, and regulatory validation, but the trajectory is clear: the future of vascular research will be shaped by platforms that recapitulate human biology with both fidelity and purpose.
